# Biomarker potential of lncRNA GNAS-AS1 in osteosarcoma prognosis and effect on cellular function

**DOI:** 10.1186/s13018-021-02611-2

**Published:** 2021-07-28

**Authors:** Zhanhu Mi, Yanyun Dong, Zhibiao Wang, Peng Ye

**Affiliations:** 1 Department of Trauma Orthopedics, General Hospital of Ningxia Medical University, 804 South Shengli Street, Yinchuan, Ningxia 750004 People’s Republic of China; 2 Operating Room, General Hospital of Ningxia Medical University, Yinchuan, Ningxia 750004 People’s Republic of China; 3 Department of Orthopedics, Rizhao Central Hospital, Rizhao, Shandong 276000 People’s Republic of China

**Keywords:** Osteosarcoma, lncRNA GNAS-AS1, Prognosis, Proliferation, Migration, Invasion

## Abstract

**Background:**

Osteosarcoma (OS) is a type of bone cancer that occurs in children and adolescents at a rate of 5%. The purpose of this study is to explore the lncRNA GNAS-AS1 expression profile, prognosis significance in OS, and biological effect on OS cell function.

**Methods:**

One hundred eight pairs of tissues were collected, and OS cell lines were purchased. lncRNA GNAS-AS1 expression in these tissues and cells were analyzed by qRT-PCR. Clinical data were analyzed using chi-square tests, Kaplan-Meier curves (log-rank test), and Cox regression. CCK-8 and transwell assay were conducted to analyze the effect of lncRNA GNAS-AS1 on cell proliferation, invasion, and migration. The downstream miRNA was presumed.

**Results:**

The expression of lncRNA GNAS-AS1 was significantly increased in OS cells and tissues, and related to Enneking staging and distant metastasis. Patients with high lncRNA GNAS-AS1 expression represented shorter overall survival and was an independent prognostic predictor of OS. LncRNA GNAS-AS1 knockdown inhibited cell proliferation, migration, and invasion by regulated miR-490-3p partly at least.

**Conclusions:**

LncRNA GNAS-AS1 can be used as a prognostic indicator and its inhibition suppress the development of OS, suggesting its value as novel therapeutic strategies in OS.

## Introduction

Characterized by mineralizing of osteoid with multinucleated cells, osteosarcoma (OS) is the most common tumor in bone tissues among children and adolescents [1, 2]. The improvement in survival of osteosarcoma has been notable on some levels, for instance, 3D tumor simulation which creates precise anatomical visualization has added benefits for radiologists, and facilitated diagnoses and surgical planning [3, 4]. Despite treatment of localized OS adhering to accepted standards, OS recurs in 30% to 40% of patients within 2–3 years of initial treatment. Recurrence usually carries a poor prognosis, and survival after recurrence is greatly reduced [5]. Till now, the biological characteristic of OS has been well understood, but the molecular mechanisms of OS origination, metastasis, or chemoresistance remain fuzzy in large regions [6]. As defined by translational medicine, the advances in basic science research would benefit the effective translation into new approaches for prevention, diagnosis, and treatment of disease for improving health [7]. Thus, new candidate molecules as prognostic biomarkers are expected to be developed, which may profoundly improve clinical outcomes for patients with OS.

Encouragingly, some long non-coding RNAs (lncRNAs) have been reported to serve as predictive, prognostic, and therapeutic biomarkers of human cancers recently, since they are usually aberrantly expressed in disease tissues and promote or inhibit tumor development, progression, and metastasis [8–11]. Altering from the perception as ‘junk’ transcriptional products, lncRNAs have been recognized to play cell-function regulatory roles and mediate cellular processes including transcription, post-transcriptional modifications, and signal transduction [12]. In OS, lncRNAs participate in cell invasion, migration, proliferation, apoptosis, and drug resistance [13–15], and offer new means for prognosis and therapy of OS [16]. LncRNA GNAS-AS1 was reported to facilitate nasopharyngeal carcinoma, ER+ breast cancer, and lung cancer cell progression [17–19]. Though LncRNA GNAS-AS1 was screened as dysregulated lncRNA in OS [20], but its role in OS has not been discussed.

In this study, the expression level of lncRNA GNAS-AS1 in OS would be determined. Based on the aberrant expression level, the potential value as prognosis predictor was accessed. The effect of lncRNA GNAS-AS1 interference on OS cellular function was analyzed, and the underly mechanism was preliminarily investigated.

## Materials and methods

### Patients and tumor samples

This study included frozen biopsy samples of 108 patients (age range 8–48 years, mean 16.7 years) who were diagnosed OS at General Hospital of Ningxia Medical University from March 2008 to December 2015. They were followed up by telephone survey at least every 3 months after completion of surgery for 2 years, and then every 6 months for at least 5 years. Two pathologists reviewed the collected biopsy specimens. This retrospective study was approved by the Institutional Review Board of General Hospital of Ningxia Medical University. Written informed consent was obtained from each participant according to the Declaration of Helsinki.

### Cell lines culture and transfection

Four human OS cell lines (MG-63, Saos-2, SW 1353, U-2 OS) and one normal osteoblast cell line, hFOB 1.19 were obtained from the National Collection of Authenticated Cell Cultures (Shanghai, China). MG-63, Saos-2, and SW 1353 cells were maintained in Dulbecco’s modified Eagle medium (DMEM, Gibco, cat# 11965092, USA), U2 OS cells in McCoy’s 5A medium (Gibco by Life Technologies, cat# 16600082), and hFOB 1.19 cells in a mixture of DMEM and Ham F12 medium (1:1 ratio, Gibco). The cell culture environment was in a humid incubator.

The siRNAs (si-GNAS-AS1 and si-control) were delegated to Ribobio (Guangzhou, China) for design and synthesis. The miR-490-3p inhibitor (anti-miR) and corresponding negative control (anti-NC) were also synthesized by Ribobio (Guangzhou, China). Lipofectamine 2000 (Invitrogen, USA) was utilized to complete the transfection and qRT-PCR was utilized to evaluate the transfection efficiency.

### RNA isolation and quantitative real-time PCR (qRT-PCR)

Total RNA was extracted via TRIzol reagent (Ambion by Life Technologies, USA) from the tissues and cell lines. The cDNA for lncRNA GNAS-AS1 was generated using lncRcute lncRNA First-Strand cDNA Synthesis kit (with gDNase) (Tiangen, China) and the quantification was performed by lnRcute lncRNA SYBR Green kit (Tiangen, China). miRcute miRNA First-Strand cDNA Synthesis kit and miRcute Plus miRNA qPCR Kit (SYBR Green; Tiangen, China) were purchased for the quantification of miR-490-3p expression level. GAPDH or U6 was employed as the internal control for quantification of lncRNA GNAS-AS1 and miR-490-3p levels. The relative expression of RNAs was calculated by the 2^−ΔΔCt^ method.

### Cell proliferation assay by cell counting kit-8 (CCK-8)

Cell proliferation of MG-63 and SW 1353 cells was analyzed with a CCK-8 assay. Cells were incubated in a 96-well plate (2 × 10^3^ cells/well) and transfected with the indicated plasmid(s) for 24 h. Subsequently, CCK-8 solution (10 μL) was added to each well every 24 h for 72 h, and the cells were further incubated for 2 h. The absorbance was measured at 450 nm, and the reference absorbance at 630 nm with a SpectraMax 190 microplate reader (Molecular Devices, USA). Three repeated experiments were performed.

### Transwell assay to determine cell migration and invasion assay

MG-63 and SW 1353 cells were trypsinized and resuspended with medium without serum. Transwell insert for invasion assay was precoated with Matrigel Matrix (Corning, USA). The cells were plated at a density of 5 × 10^4^ cells/well in the upper compartment of transwell insert (Corning, USA). The chemoattractant in the lower compartment was 10% FBS. After a 24-h incubation, the uninvaded cells were removed, while the passed cells were fixed with methanol for 1 h and then stained with 0.1% crystal violet. Five random fields of vision were taken under the microscope and the stained cells were counted manually.

### Target miRNA prediction and luciferase reporter assay

LncBase Predicted v.2 and lncRNASNP2 were used to predict the potential miRNAs targeting lncRNA GNAS-AS1. pLHCX retroviral vector containing wild-type lncRNA GNAS-AS1 (WT- GNAS-AS1) or mutated one (MUT-GNAS-AS1) generated by site-directed mutagenesis were purchased from Ribobio (Guangzhou, China). MG-63 cells were co-transfected with wild-type or mutated lncRNA GNAS-AS1 reporter plasmids, and with miR-490-3p mimics or inhibitor or corresponding negative control. Twenty-four hours post-transfection, the luciferase activity was assayed using the 100T Promega E1910 Dual Luciferase Reporter Assay System (Promega, USA) following the manufacturer’s instruction.

### Statistical analysis

The data were represented as mean with SD. Student’s *t* test, one-way, or two-way ANOVA was used to calculate the significance of the data. Clinical data were analyzed using chi-square tests, Kaplan-Meier curves (log-rank test), and Cox regression. Chi-square tests were for the access of association between lncRNA GNAS-AS1 and the clinical parameters. Kaplan-Meier curves were plotted to show a direct comparison visually. Next, the statistical significance of each prognostic factor was assessed by comparing likelihood ratios from multivariate Cox analysis. Correlation analysis between lncRNA GNAS-AS1 and miR-490-3p expression was performed by Pearson's correlation coefficient (Pearson’s r) with a two-tailed test. *P* < 0.05 was considered significant.

## Results

### Upregulation of lncRNA GNAS-AS1 in OS tissues and cell lines

**Fig. 1:**
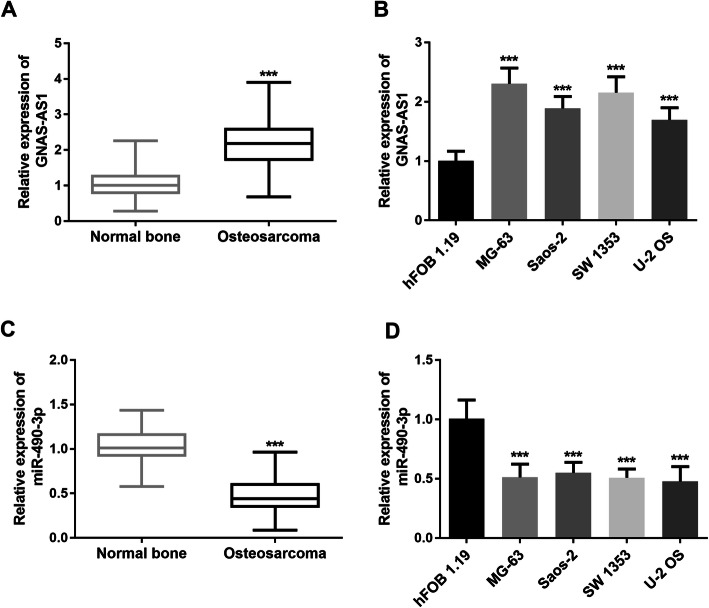
LncRNA GNAS-AS1 is downregulated in osteosarcoma tissues and cell lines. **A** The relative expression of lncRNA GNAS-AS1 in osteosarcoma tissues and corresponding normal bone tissue samples (*n* = 108). **B** LncRNA GNAS-AS1 expression in osteosarcoma cell lines (MG-63, Saos-2, SW 1353, U-2 OS) and one normal osteoblast cell line hFOB 1.19. **C** The relative expression of miR-490-3p in osteosarcoma tissues and corresponding normal bone tissue samples (*n* = 108). **D** miR-490-3p expression in osteosarcoma cell lines (MG-63, Saos-2, SW 1353, U-2 OS) and one normal osteoblast cell line hFOB 1.19. Data are all obtained from qRT-PCR. ^***^P < 0.001

To obtain the expression profile of lncRNA GNAS-AS1 in OS, qRT-PCR was utilized to determine its level in OS tissues, the matched normal tissues, OS cell lines, and normal osteoblast cell line. Compared to the normal tissues, lncRNA GNAS-AS1 was found with an ascendant expression level in OS tissues (*P* < 0.001, Fig. 1A). Similarly, expression of lncRNA GNAS-AS1 in OS cell lines was significantly higher than that in osteoblast cell line hFOB1.19, especially in MG-63 and SW 1353 cell line which would be used in the subsequent test (*P* < 0.001, Fig. 1B). On the contrary, the expression level of miR-490-3p in OS tissues and cell lines was significantly decreased compared with normal ones (*P* < 0.001, Fig. 1C, D).

### Relationships between lncRNA GNAS-AS1 and clinicopathologic factors

The correlations between lncRNA GNAS-AS1 and clinicopathological variables of OS patients are presented in Table 1. According to the mean value of lncRNA GNAS-AS1 expression, the OS patients were divided into high expression group (*n* = 59) and low expression group (*n* = 49). From Table 1, overexpression of lncRNA GNAS-AS1 was significantly related to Enneking stage (*P* = 0.018) and distant metastasis (*P* = 0.030) in OS patients. While no significant differences were obtained between lncRNA GNAS-AS1 expression and other parameters (all *P* > 0.05).

**Table 1:** Correlation between lncRNA GNAS-AS1 expression levels and clinical features in OS patients

Parameter	Cases no. (*n* = 108)	LncRNA GNAS-AS1 expression		*P*
		Low (*n* = 49)	High (*n* = 59)	
Gender				0.239
Male	55	28	27	
Female	53	21	32	
Age (years)				0.586
< 19	52	25	27	
≥ 19	56	24	32	
Enneking stage				0.018*
I-IIA	44	26	18	
IIB-III	64	23	41	
Tumor size				0.069
< 8 cm	58	31	27	
≥ 8 cm	50	18	32	
Location of lesion				0.957
Axial bone	51	23	28	
Others	57	26	31	
Distant metastasis				0.030*
Absence	65	35	30	
Presence	43	14	29	

### Increase of lncRNA GNAS-AS1 correlates with poor prognosis in OS patients

**Fig. 2:**
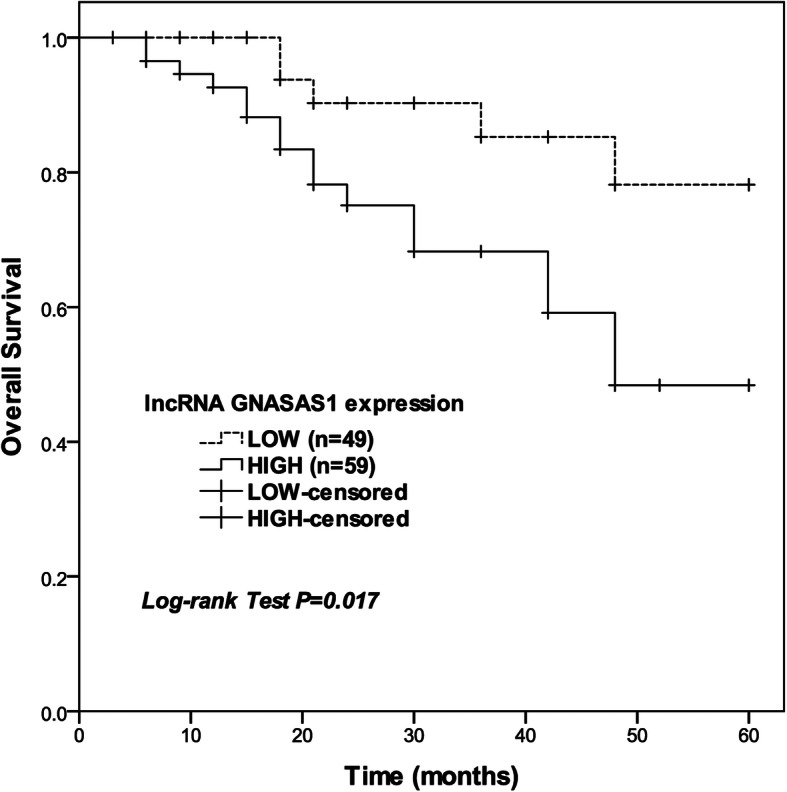
Overall survival times were plotted via Kaplan-Meier survival curves and analyzed by log-rank tests (log-rank *P* = 0.017)

The discrimination of overall survival between high and low lncRNA GNAS-AS1 expression group was assessed by Kaplan-Meier plots with 95% confidence intervals (log-rank test *P* = 0.017, Fig. 2). High lncRNA GNAS-AS1 expression group showed shorter overall survival (44.1 ± 3.01) than low lncRNA GNAS-AS1 expression group (54.0 ± 2.5). Moreover, to determine the prognostic significance contributed by lncRNA GNAS-AS1 expression after accounting for other important prognostic factors, multivariate Cox proportional hazards regression was used to assess the impact of various prognostic factors. As shown in Table 2, lncRNA GNAS-AS1 expression were identified as significant and independent prognostic variable (HR = 4.519, 95%CI = 1.473–13.865, *P* = 0.008).

**Table 2:** Multivariate Cox analysis of prognostic factors in OS patients based on the overall survival

Parameter	Multivariate analysis		
	HR	95%CI	*P*
LncRNA GNAS-AS1	4.519	1.473–13.865	0.008
Gender	2.025	0.732–5.605	0.174
Age	1.167	0.473–2.881	0.737
Tumor size	1.671	0.591–4.726	0.333
Location of lesion	2.371	0.875–6.419	0.089
Enneking stage	8.172	0.973–68.664	0.053
Distant metastasis	5.291	1.455–19.242	0.011

### Knockdown of lncRNA GNAS-AS1 inhibited proliferation, invasion, and migration of human OS cells

**Fig. 3:**
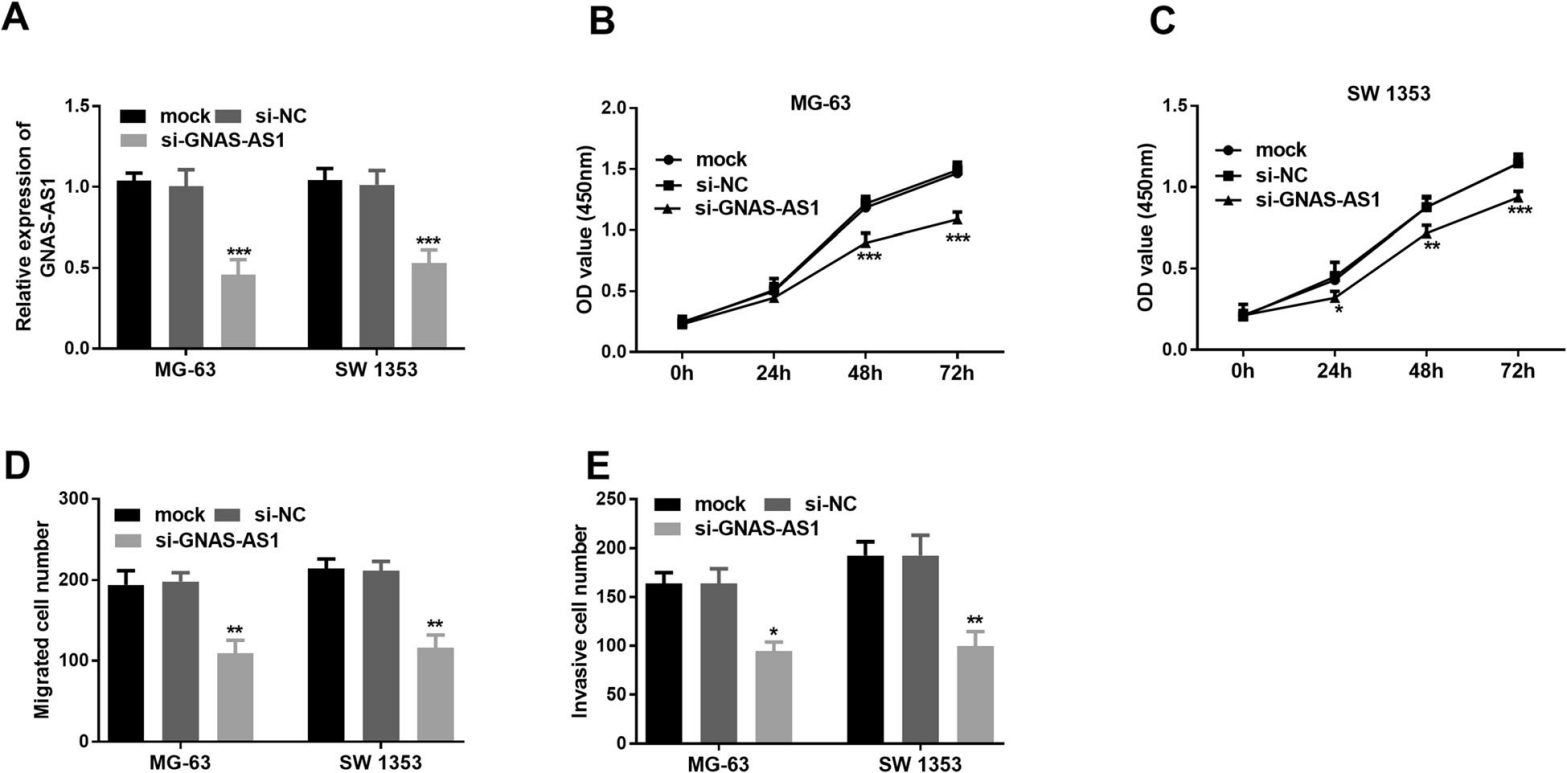
Downregulation of lncRNA GNAS-AS1 inhabited OS cell proliferation, migration, and invasion. **A** MG-63 and SW 1353 cells were transfected with the si-GNAS-AS1 or si-NC. **B**, **C** Cell proliferation was monitored to be suppressed by si-GNAS-AS1. **D** Cell migration was analyzed by transwell assay. **E** Cell invasion was measured using modified transwell assay. ^*^P < 0.05, ^**^P < 0.01, ^***^P < 0.001

To clarify the roles of lncRNA in tumorigenesis and the progression of OS, small interference RNA (si-GNAS-AS1) was used to knock down lncRNA GNAS-AS1 in MG-63 and SW 1353 cells. siRNA exhibited significant inhibition potency in these two cell lines (*P* < 0.001, Fig. 3A). Correspondingly, we found an inhibition of cell proliferation with lncRNA GNAS-AS1 knockdown in these two OS cells (*P* < 0.05, Fig. 3B, C). lncRNA GNAS-AS1 has also been reported to promote cell migration and invasion in ER+ breast cancer [18]; thus, we assayed for the effects of lncRNA GNAS-AS1 knockdown on cell migration and invasion in OS cells using transwell assay. The cell invasion and migration of MG-63 and SW 1353 cells with lncRNA GNAS-AS1 interference was suppressed (*P* < 0.05, Fig. 3D, E).

### miR-490-3p was target miRNA of lncRNA GNAS-AS1

**Fig. 4:**
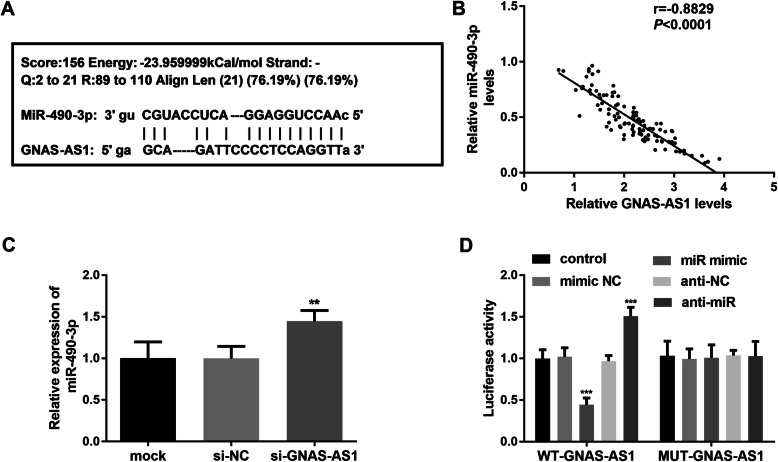
miR-490-3p was downstream miRNA of lncRNA GNAS-AS1. **A** Binding sites for lncRNA GNAS-AS1 in the miR-490-3p 3′UTR were predicted using lncRNASNP2. **B** Expression of lncRNA GNAS-AS1 and miR-490-3p is negatively correlated. **C** Expression of miR-490-3p was raised when lncRNA GNAS-AS1 was inhibited. **D** pLHCX retroviral vector with wild-type lncRNA GNAS-AS1 (WT-GNAS-AS1) and mutated one (MUT-GNAS-AS1) were constructed, and co-transfected into MG-63 cells with the miR-490-3p mimic or inhibitor. The relative luciferase activity was measured. ^**^P < 0.01, ^***^P < 0.001

LncBase predicted v.2 and lncRNASNP2 showed miR-490-3p was one of the lncRNA GNAS-AS1 target miRNAs (Fig. 4A). Since miR-490-3p has shown a decreased expression in OS, and the expression level was strongly but negatively correlated with the expression of lncRNA GNAS-AS1 by Pearson’s correlation coefficient (*r* = − 0.8829, *P* < 0.001, Fig. 4B). And inhibition of lncRNA GNAS-AS1 can lead an increase in the expression of miR-490-3p (*P* < 0.01, Fig. 4C). To verify this further, luciferase assay was used to analyze the effect of miR-490-3p mimic or inhibitor on the luciferase activity of WT-GNAS-AS1 or MUT-GNAS-AS1. The luciferase activity of wide-type constructs exhibited significant reduction under miR-490-3p mimic and rise under miR-490-3p inhibitor (*P* < 0.001, Fig. 4D). Whereas the luciferase activity of MUT-GNAS-AS1 has no alteration with the existence of miR-490-3p mimic or inhibitor.

**Fig. 5:**
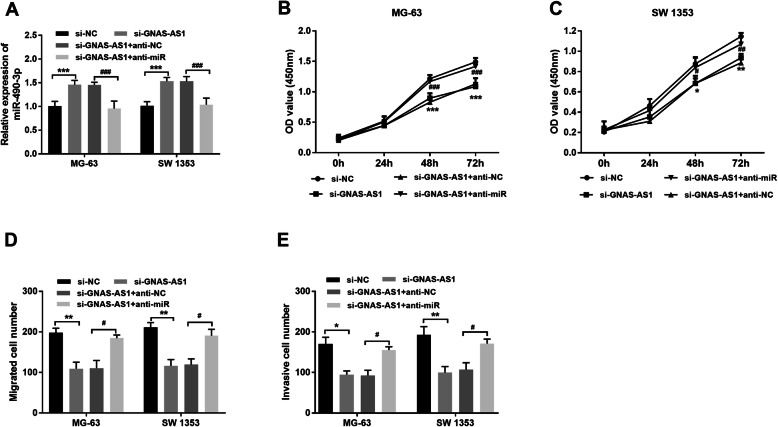
miR-490-3p counteracted the cellular function of lncRNA GNAS-AS1. **A** The effect of transfection was confirmed by qRT-PCR. **B**, **C** miR-490-3p downregulation can offset the suppression of proliferation by si-GNAS-AS1. **D** miR-490-3p downregulation can neutralize the inhibition of migration by si-GNAS-AS1. **E** miR-490-3p downregulation can abolish the repression of cell invasion by si-GNAS-AS1. ^*^P < 0.05, ^**^P < 0.01, ^***^P < 0.001 (si-GNAS-AS1 vs. si-NC). ^#^*P* < 0.05, ^##^*P* < 0.01, ^###^*P* < 0.001 (si-GNAS-AS1 + anti-miR vs. si-GNAS-AS1 + anti-NC)

Furtherly, a rescue experiment was performed. The transfection efficiency was confirmed by miR-490-3p expression (*P* < 0.001, Fig. 5A). In the followed CCK8 assay, cell proliferation reduced by lncRNA GNAS-AS1 knockdown can be restored by inhibition of miR-490-3p (*P* < 0.05, Fig. 5B, C). Furthermore, cell migration and invasion detected by transwell assay were increased again when si-GNAS-AS1 and miR-490-3p inhibitor co-existed (*P* < 0.05, Fig. 5D, E). Rescue experiments suggest that miR-490-3p can reverse the cellular effect of lncRNA GNAS-AS1.

## Discussion

Despite cancer is usually common in adults than children and adolescents [21], OS is characteristic of a bimodal age distribution with an adolescent (0–20 years old) and an elderly (centered at age 75 years) peak in incidence [22]. Considering the majority of children and adolescents involved in high-grade OS whose remaining life expectancy is crucial, the prediction of prognosis becomes very important [23]. Besides, the prognosis for patients with metastatic OS is much poorer than for those with only primary OS [24]. Therefore, new prognosis predictor should be developed to improve the outcome estimate.

The prognosis value of lncRNAs has been gradually revealed recently, such as lnc-HOST2, lncRNA CBR3-AS1, or lncRNA TP73-AS1 [25–27]. Since lncRNA GNAS-AS1 has been characterized as one lncRNA significantly associated with OS patients’ survival by bioinformatics, this study focused on the clinical significance of lncRNA GNAS-AS1 in the OS cases of our institution. Firstly, the expression of lncRNA GNAS-AS1 in 108 OS tissues and a panel of cell lines were detected by qRT-PCR, and was found to be upregulated in tumor tissues and cancerous cell lines. In line with this, upregulation of lncRNA GNAS-AS1 was also found in nasopharyngeal cancer, breast cancer, and non-small cell lung cancer [17–19]. Furtherly, the patients were divided into two groups based on the mean expression value of lncRNA GNAS-AS1, and the association between the expression and clinical parameters was accessed by chi-square test, which showed the high expression of lncRNA GNAS-AS1 was related to high Enneking stage and positive distant metastasis. This relationship suggests to us the potential prognosis value of lncRNA GNAS-AS1. In order to verify the prognosis value, Kaplan-Meier curves were plotted to directly display the distinction of the overall survival between patients with high lncRNA GNAS-AS1 expression and low expression, and multivariate Cox analysis was introduced to access the statistical significance of lncRNA GNAS-AS1 as prognostic factor. The result showed lncRNA GNAS-AS1 was identified as an independent prognosis factor for overall survival.

Elucidation of the molecular basis on which lncRNAs affect OS development and progression is essential to establish better treatment options [1, 28]. Recently, lncRNAs have been identified that participate in OS progression, including cancerous cell growth, metastasis, and apoptosis [29–31]. LncRNA GNAS-AS1 was an important gene related to the formation and progression of nasopharyngeal carcinoma and breast cancer [17, 18]. In this study, the effect of lncRNA GNAS-AS1 interference on OS cellular growth and metastasis was explored. By CCK-8 assay, the proliferation was found to be suppressed by lncRNA GNAS-AS1 knockdown, and by transwell assay, the migration and invasion of OS cells were inhibited by lncRNA GNAS-AS1 downregulation. These suggest that lncRNA GNAS-AS1 plays a role in promoting OS development, which can also be a new potential therapeutic target for OS.

LncRNAs can act as microRNA sponge to modulate gene expression, which involves various ncRNAs in interconnected competitive regulatory interactions, known as competing endogenous RNA (ceRNA) network [32]. Thus, the aberrant expression of lncRNA could influence the downstream miRNA directly, culminating in development and progression of cancer. To find the possible target miRNAs of lncRNA GNAS-AS1, LncBase Predicted v.2 and lncRNASNP2 were used to search, and miR-490-3p got our attention since its decreased expression and prognosis value in OS [33]. To confirm the binding relationship of miR-490-3p and lncRNA GNAS-AS1, the expression of miR-490-3p under lncRNA GNAS-AS1 interference was detected to be increased, and the expression profile of these two RNA was negatively correlated. Luciferase reporter assay and rescue experiment furtherly confirmed lncRNA GNAS-AS1 act as ceRNA of miR-490-3p to exert its cellular function.

The limitation of study population just in our single organization is disadvantage of this study. A larger number of subjects would be needed to validate lncRNA GNAS-AS1 values in OS prognosis. Moreover, in vivo experiments related to the cellular function of lncRNA GNAS-AS1 are needed to explore the deep mechanism. Future research would benefit from evaluating lncRNA GNAS-AS1 clinical significance in larger samples. In addition, it would be useful for development of new treatment tool to explore the thorough mechanism of lncRNA GNAS-AS1 effecting on the cellular function.

## Conclusion

In conclusion, lncRNA GNAS-AS1 was with an increased expression in OS, and this overexpression was associated with unfavorable pathological characteristics and shorter overall survival. Knockdown of lncRNA GNAS-AS1 inhibits cell growth, migration, and invasion. LncRNA GNAS-AS1 has the potential to be used as an independent prognostic factor and new molecular therapeutic approach. Finally, further studies are desirable to validate the accuracy of lncRNA GNAS-AS1 as prognosis biomarker in a series of patient studies in order to determine the accuracy in a clinical setting.

## Data Availability

Corresponding authors may provide data and materials.
